# Knowledge of oral health, health literacy, and oral-health behaviors among pharmacy students in Japan

**DOI:** 10.1186/s40780-026-00614-x

**Published:** 2026-07-31

**Authors:** Tomiko Sunaga, Rie Yokota, Tomofumi Watanabe, Wataru Sakurada, Hiroyuki Terakado

**Affiliations:** 1 Department of Clinical Epidemiology, Division of Applied Pharmaceutical Education and Research, Hoshi University, 2-4-41 Ebara, Shinagawa-ku, Tokyo, 142-8501 Japan; 2 Department of Medical Communication, School of Pharmacy and Pharmaceutical Sciences, Hoshi University, Tokyo, Japan; 3 Faculty of Pharmaceutical Sciences, Health Sciences University of Hokkaido, Hokkaido, Japan

**Keywords:** Health literacy, Oral care, Pharmacy students, Preventive dental visit

## Abstract

**Background:**

Community pharmacies are expected to play a role in oral health promotion and disease prevention. However, knowledge of oral health and oral-health behaviors among Japanese pharmacy students remains unclear. The purpose of this study was to assess the knowledge of oral care (OC) and health literacy (HL) among Japanese pharmacy students and to identify factors associated with their preventive dental visits.

**Methods:**

Pharmacy students at Hoshi University were enrolled. Questions from the Oral Care Ambassador certification textbook were selected to assess knowledge regarding 13 broad domains. HL was assessed using the short version of the European HL Survey Questionnaire. A logistic regression model was constructed using preventive dental visits as the outcome variable.

**Results:**

The proportion of students with knowledge regarding assistance and home healthcare for OC and dysphagia/rehabilitation was small, and the scores were low on several practical HL items. Students who preventively visited a dentist showed significantly higher rates of dental floss and interdental brush use compared to those who did not.

**Conclusions:**

This study suggests that pharmacy students before clinical practice may lack knowledge of home healthcare and dysphagia/rehabilitation of OC, and that their practical HL skills in clinical settings are low. These findings may provide insights for addressing challenges in the teaching methods for OC and HL among pharmacy students in the future.

**Supplementary Information:**

The online version contains supplementary material available at https://doi.org/10.1186/s40780-026-00614-x.

## Background

Healthcare professionals and public-health practitioners advocate that community pharmacies play a greater role in promoting oral health and preventing disease [1–3]. In the UK, the public recognizes community pharmacies as the appropriate providers of oral-health information [4]. In contrast, in Australia, although pharmacies receive several oral-care consultations, > 80% of the pharmacy staff reported a lack of patient-facing oral health-education resources and access to oral-health information for their own education [5]. Consequently, countries are exploring ways in which pharmacists engage in oral care (OC). In Japan, starting in April 2024, students entering pharmacy programs are subject to the “Model Core Curriculum for Pharmacy Education” [6], which incorporates learning about OC that considers the patient’s condition and activities that contribute to maintaining and improving the health such as oral health of individual residents in the community. Therefore, the establishment of effective learning methods is an urgent priority.

Oral health is closely linked to the overall health; in particular, poor oral hygiene is associated with an increased risk of developing systemic diseases such as diabetes and cardiovascular disease [7, 8]. Improvement of health literacy (HL) of the public is desirable for promoting health and reducing the risk of developing these systemic diseases. HL is defined as “the knowledge, motivation, and ability to access, understand, evaluate, and apply health information to make judgments and decisions regarding healthcare, disease prevention, and health promotion in order to maintain or improve quality of life throughout one’s lifetime [9]. General HL is not usually considered specifically in the context of oral health. However, because oral health is an integral component of overall health, HL research should include behaviors and decision-making related to oral health [10]. Healthcare professionals also play an important role in supporting improvement in people’s HL [11]. Therefore, pharmacy students—who will eventually serve as pharmacists and support improvements in people’s health—are expected to develop HL as a broad concept that extends beyond oral health alone.

College students, who are beginning to take personal responsibility for their oral health habits and regular dental checkups, are in a transitional phase of developing lifelong healthy habits and thus represent a key demographic for promoting oral health. The adult Japanese Cohort Study reported that participants with high HL had better oral health outcomes [10]. In addition, previous studies have reported that college students with greater awareness of oral health tend to practice healthier oral health habits [12]. Therefore, it is important for pharmacy students to enhance their HL and knowledge of oral health and practice good OC from the time of their undergraduate education. However, because students in healthcare fields are often assumed to possess specialized knowledge, few studies on the HL and oral health behaviors of pharmacy students in Japan have been reported. This study aimed to (1) assess the knowledge of OC and HL among Japanese pharmacy students and (2) explore the factors, including HL and oral health behaviors associated with their preventive dental visits.

Oral HL and Health Integration Framework is designed to recognize the complex factors that contribute to both integration and HL and ultimately to health equity. This framework emphasizes that HL plays a crucial role in promoting the integration of oral health and systemic health, and in supporting health-related decision-making [13]. Therefore, as higher levels of HL are expected to support informed decision-making and engagement in preventive health behaviors, pharmacy students with higher general HL may be more likely to adopt preventive oral health behaviors, including regular preventive dental visits.

## Methods

### Study design and population

**Fig. 1:**
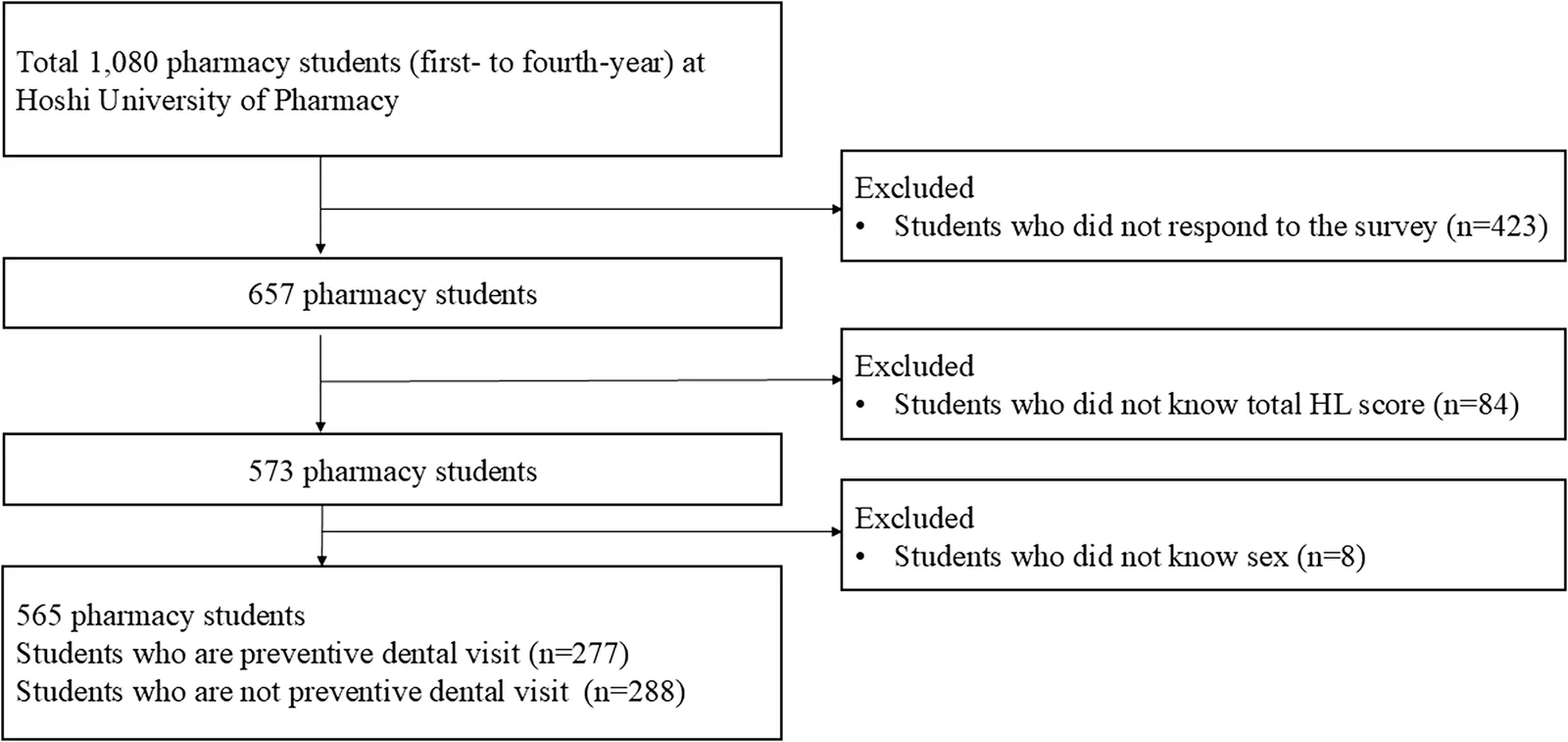
Flowchart of this study. Of the 1,080 pharmacy students enrolled, 657 responded to the questionnaire. Of these, 573 students completed all health literacy items. After we excluded 8 students who did not know sex, 277 students visited a dentist for preventive care, while 288 did not visit them

This cross-sectional exploratory study evaluated pre-clinical practice knowledge of OC and HL among first- to fourth-year pharmacy students enrolled at Hoshi University of Pharmacy. Participants were asked to complete a computerized questionnaire, and those with missing values were excluded. Figure 1 shows the flowchart for this study. Data was collected from July 2025 to December 2025. Researchers explained the purpose to the participants, respondents were anonymous, and all participants agreed to participate before taking the survey. The researchers provided participants with written explanations regarding the purpose of the research either at the beginning or the end of the lecture, and clearly stated that their participation would not affect their academic performance or credit acquisition for the subjects taught by the researchers and that there would be no disadvantages for not responding. Students answered the questionnaire by scanning the QR code attached to the document. This study protocol followed the ethical guidelines established by the Ethical Guidelines for Medical and Biological Research Involving Human Subjects. This study was approved by the Hoshi University Research Ethics Review Board (approval number: 2025-05).

## Assessment of knowledge and HL

A total of 20 questions from the Oral Care Ambassador certification textbook were selected to assess knowledge regarding 13 broad domains (number of questions) [14], including the definition of OC (2 questions), role and effects of OC (2 questions), OC characteristics (1 question), oral health and preventive dentistry (3 questions), oral structure (1 question), oral function (2 questions), periodontal disease (1 question), xerostomia/stomatitis (1 question), OC timing/frequency (1 question), Choosing and using a toothbrush (1 question), OC assistance/nursing (2 questions), dysphagia/rehabilitation (2 questions), and lifestyle habits/medications/bleeding (1 question). The 13 broad domains were selected by researchers who were faculty members in the School of Pharmacy. Questions were answered as “Yes” or “No,” and performance was evaluated based on the accuracy rate. The categories and questions are listed in Additional File 1. For domains with multiple questions, the results are presented as the average accuracy rate. No standardized scale has been established for assessing pharmacy students’ knowledge of OC in Japan. Therefore, this study utilized the textbook for the “Oral Care Ambassador” certification exam provided by the Japanese Society of Oral Care, which pharmacy students use as a reference for obtaining their Oral Care Ambassador certification. Since the Oral Care Ambassador exam is administered via computer-based testing (CBT), the questionnaire was designed to require “Yes” or “No” responses. Therefore, the validity of the selected items and their content used to assess pharmacy students’ knowledge of OC has not been verified.

The European Health Literacy Survey Questionnaire (HLS-EU-Q47) [15] developed by Sørensen et al. is available in multiple languages, and the shortened Japanese version (HLS-Q12) [16], which consists of three domains: Healthcare (HC), Disease Prevention (DP), and Health Promotion (HP), was used to assess HL. Prior approval was obtained from the authors for the use of the Japanese version. One of the most widely used measures of oral HL is the HL in Dentistry Scale (HeLD) [17]. Oral HL, unlike HL (which is a broader concept) refers specifically to the ability to understand and use information related to oral health in order to engage in appropriate oral-health behaviors [18]. However, because oral health is part of overall health and this study focused on pharmacy students as future healthcare professionals, general HL was assessed using the Japanese version of the HLS-Q12, which has been validated for reliability and validity. Responses ‘very difficult,’ ‘difficult,’ ‘easy,’ and ‘very easy,’ were scored as 1, 2, 3, and 4, respectively, with a higher score indicating higher HL. In addition, participants had the option of selecting ‘do not know,’ which was categorized as missing. The HLS-Q12 total score ranges from 12 to 48 and was treated as a continuous variable.

We investigated age, sex, grade level, prior OC education, presence of healthcare professionals in the respondent’s immediate environment, oral-health behaviors, knowledge of OC, and HL. Regarding preventive dental visit, the question explicitly stated “visited a dentist for purposes other than treatment (e.g., cleaning)” to clearly identify preventive dental visits.

### Statistical analysis

Participant characteristics were described as numerical values and percentages for categorical variables, and as mean ± standard deviation for continuous variables. The correct response rate for OC questions was examined for trends according to grade level using the Cochran–Armitage test. We defined the “preventive dental visit group” as pharmacy students who had visited a dentist for purposes other than treatment (such as cleaning) within the past year. Among the participants, 84 selected “do not know,” in the HL questionnaire; these responses were treated as missing. In addition, eight participants did not provide sex information. After excluding participants with missing data, 565 participants were included in the final analysis.

The required sample size was calculated using G*Power 3.1.9.7 [19, 20]. A previous survey reported that approximately 40% of people who receive dental checkups at least once a year use interdental brushes or mouthwash, while only approximately 20% of those who do not receive checkups use them [21]. Therefore, assuming oral-health behavior implementation rates of 40% and 20% among preventive dental care recipients and non-recipients, respectively, the required total sample size for a significance level of 0.05 and a power of 80% was estimated to be 167 participants. The adequacy of the sample size in the logistic regression analysis was evaluated using the events-per-variable (EPV) principle. This model included 277 outcome events and 8 explanatory variables, resulting in an EPV value of 34.6, which substantially exceeds the generally recommended minimum threshold of 10, indicating sufficient model stability. Participant characteristics, HL, oral health behaviors were compared between students with and without preventive dental visits. Univariate analysis was performed using the χ2 test for categorical variables and the Wilcoxon rank-sum test for continuous variables. A logistic regression model was constructed using preventive dental visit as the outcome variable. HL, sex, and oral health behaviors (such as flossing and mouthwash use) were entered as covariates. All covariates were measured in the same participants. The covariates were selected based on prior research to include general oral health behaviors [22]. Multivariate logistic regression analysis was conducted to calculate the adjusted odds ratio and 95% confidence intervals (CI). All analyses were performed using JMP Pro Version 19.0 (SAS Institute Inc., Cary, NC, USA), and statistical significance was set at two-tailed *p* < 0.05.

## Results

### Participant characteristics

Of the 1,080 pharmacy students enrolled, 657 responded to the questionnaire (response rate, 60.8%). Of these, 573 (87.2%) completed all HL items. Table 1 presents the participants’ characteristics. The average age was 20.1 ± 1.5 years, with 26.6% men, 71.5% women and 1.8% students who did not know sex. Among the participants, 60.6% had learned about OC between elementary and high school, with most learning during elementary school. Additionally, 48.4% of participants visited a dentist for preventive care in the past 1 year. The most common reasons for visiting were to continue preventive visits after completion of treatment (169 participants) and the influence of family members who regularly visited dentists (120 participants).

**Table 1:** Participants' Characteristics (n=657)

	All	First year	Second year	Third year	Fourth year
	n=657	n=171	n=174	n=153	n=159
Age, years, mean ± SD	20.1 ± 1.5	18.9 ± 0.9	19.6 ± 1.3	20.4 ± 0.7	21.9 ± 1.0
Sex, n (%)					
Female	470 (71.5)	123 (71.9)	113 (64.9)	117 (76.5)	117 (73.6)
Male	175 (26.6)	44 (25.7)	57 (32.8)	34 (22.2)	40 (25.2)
Unknown	12 (1.8)	4 (2.3)	4 (2.3)	2 (1.3)	2 (1.3)
Existence of medical pofessional among family or friends	246 (37.4)	60 (35.1)	66 (37.9)	55 (36.0)	65 (40.9)
Have you ever learned about oral care at school between elementary school and high school graduation?					
Yes	398 (60.6)	91 (53.2)	109 (62.6)	90 (58.8)	108 (67.9)
Oral health-related behaviors, n (%)					
Fluoride toothpaste	497 (75.7)	129 (75.4)	128 (73.6)	124 (81.1)	116 (73.0)
Mouthrinse	170 (25.9)	42 (24.6)	49 (28.2)	38 (24.8)	41 (25.8)
Dental floss or interdental brush	327 (49.8)	79 (46.2)	84 (48.3)	74 (48.4)	90 (56.6)
Electric toothbrush	69 (10.5)	20 (11.7)	18 (10.3)	16 (10.5)	15 (9.4)
Tooth brushing frequency (twice daily or more)	600 (91.3)	153 (89.5)	158 (90.8)	143 (93.5)	146 (91.8)
Preventive dental visit in the past 1 year	318 (48.4)	92 (53.8)	72 (41.4)	76 (49.7)	78 (49.1)

SD, standard deviation

### Knowledge of OC and oral-health behaviors

**Fig. 2:**
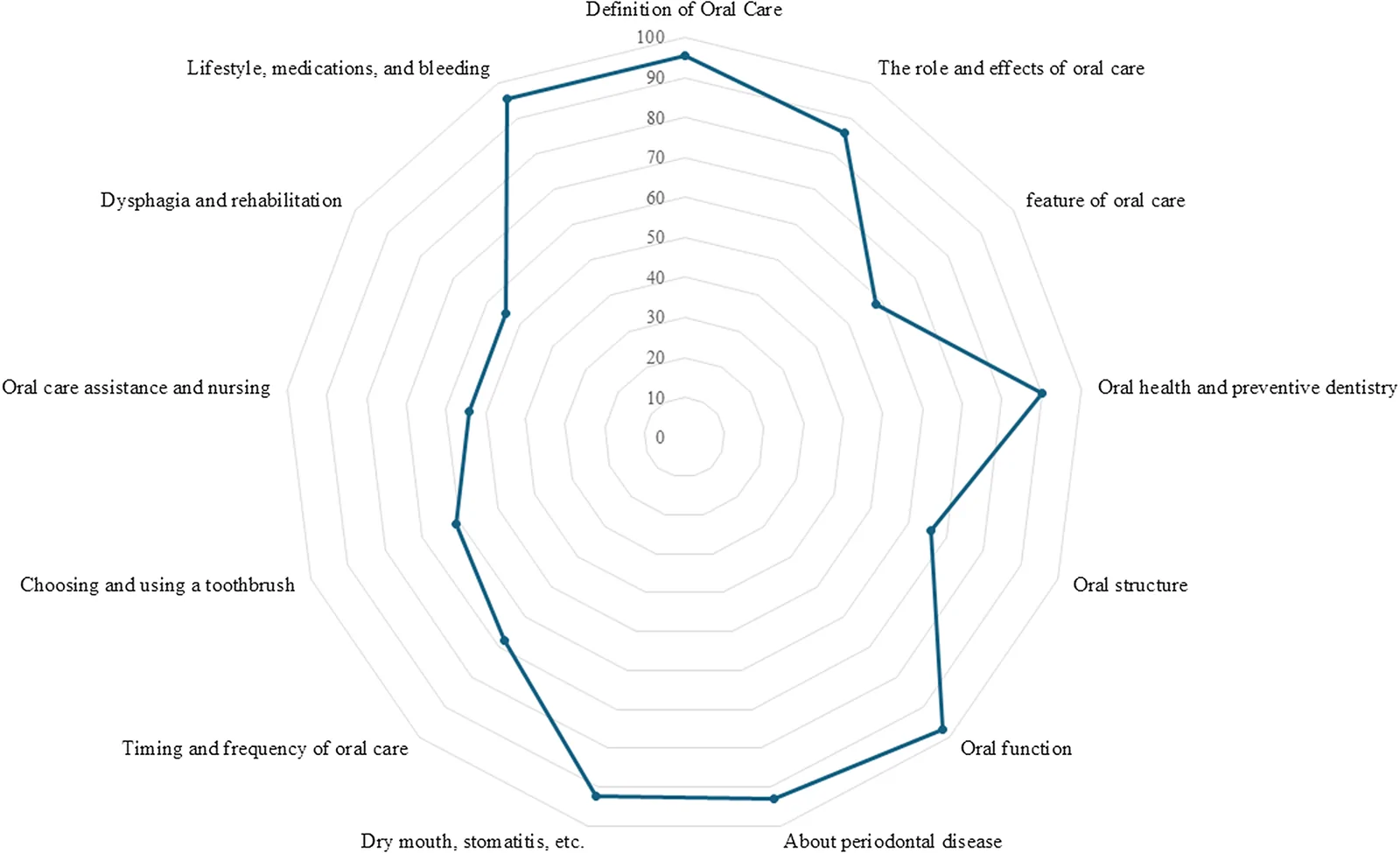
Knowledge of Oral Care. High correct answer rates are observed in the domains of oral care definition, oral care and preventive dentistry, oral function, periodontal disease, oral dryness/stomatitis, and lifestyle-related diseases/medications/bleeding. Conversely, correct answer rates are low for characteristics of oral care, mechanisms of the oral cavity, timing and frequency of oral care, toothbrush selection and usage, assistance and nursing care for oral care, and dysphagia/rehabilitation

Figure 2 shows pharmacy students’ knowledge of OC in the 13 domains. High correct answer rates were observed in the domains of definition of OC (95.5%), oral health and preventive dentistry (90.2%), oral function (97.4%), periodontal disease (93.2%), oral xerostomia/stomatitis (92.5%), and lifestyle habits /medications/bleeding (95.7%). Conversely, low correct answer rates were observed for OC characteristics (58.3%), oral structure (66.1%), OC timing and frequency (67.9%), and choosing and using a toothbrush (60.9%). Particularly, OC assistance/nursing (54.3%) and dysphagia/rehabilitation (54.3%) had rates of approximately 50%. Interestingly, the correct answer rates for eight items tended to increase with grade level (Supplementary Table 1; all p trend < 0.05).

The most common oral-health behaviors were the use of fluoride toothpaste (75.6%) and dental floss or interdental brush (49.8%). Most pharmacy students (91.3%) brushed their teeth at least twice daily (Table 1).

### HL

**Fig. 3:**
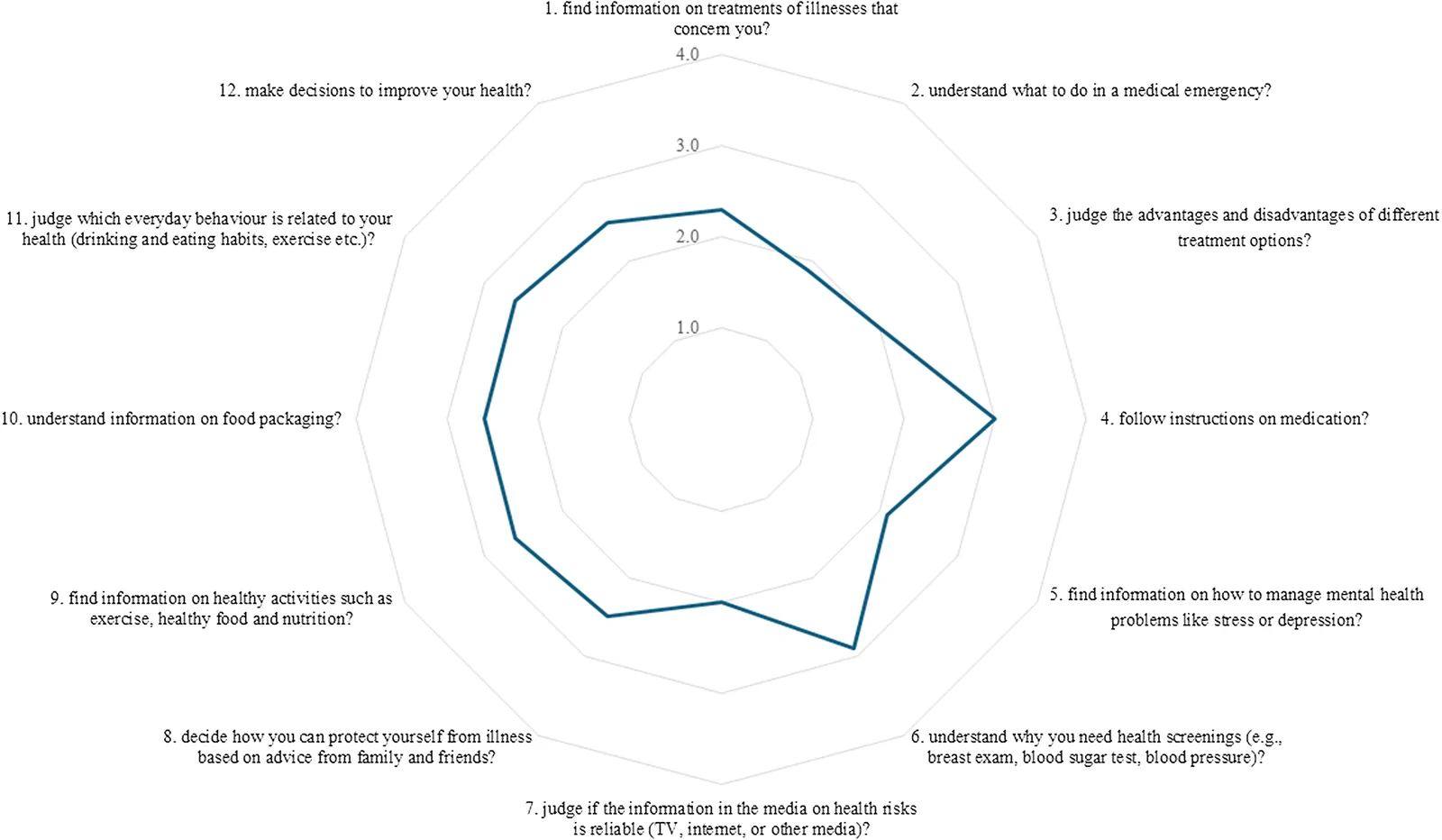
Health Literacy score of pharmacy students. Among the HC items, scores are low for “understand what to do in a medical emergency” and “judge the advantages and disadvantages of different treatment options.” Conversely, the score for “follow instructions on medication” is high. Among the DP items, scores are low for “judge if the information in the media on health risks is reliable.” Conversely, scores are high for “understand why you need health screenings” and “decide how you can protect yourself from illness based on advice from family and friends.” HP shows high scores across all items. HC, healthcare; DP, disease prevention; HP, Health Promotion

Figure 3 and Supplementary Table 2 (See Additional File 2) show the comprehensive HL scores for pharmacy students. The mean total HL score was 29.1 ± 5.5. Among the HC items, scores were low for “understand what to do in a medical emergency” and “judge the advantages and disadvantages of different treatment options.” Conversely, the score for “follow instructions on medication” was high. Regarding DP, scores were low for “judge if the information in the media on health risks is reliable.” Conversely, the scores were high for “understand why you need health screenings” and “decide how you can protect yourself from illness based on advice from family and friends.” Regarding HP, high scores were observed for all items.

### Relationship between preventive dental visit and oral-health behaviors

The association between preventive dental visits and HL or oral health behaviors is shown in Table 2. Students who visited a dentist for preventive care had significantly higher total HL scores (29.6 ± 5.8 vs. 28.6 ± 5.2, *p* = 0.04) than those who did not visit a dentist for preventive care. The preventive dental visit group also exhibited a higher rate of dental floss and interdental brush use (64.6% vs. 36.5%, *p* < 0.01) and more frequently had medical professionals among family members or friends (41.9% vs. 33.3%, *p* = 0.04). In particular, students who visited a dentist for preventive care had significantly higher HP-HL scores (10.5 ± 2.4 vs. 10.1 ± 2.2, *p* = 0.01).

**Table 2:** Association between preventive dental visit, and health literacy or oral-health behaviors (n=565)

	Preventive dental visit	No preventive dental visit	p-value
	n=277	n=288	
Total HL, mean ± SD	29.6 ± 5.8	28.6 ± 5.2	0.04
median (IQR)	29 (26, 33)	28 (25, 32)	
Health Care	9.3 ± 2.0	9.1 ± 1.9	0.13
median (IQR)	9 (8, 10)	9 (8, 10)	
Disease Prevention	9.8 ± 2.2	9.4 ± 2.1	0.08
median (IQR)	10 (8, 11)	9 (8, 11)	
Health Promotion	10.5 ± 2.4	10.1 ± 2.2	0.01
median (IQR)	11 (9, 12)	10 (8,12)	
Sex, n (%)			
Female	205 (74.0)	206 (71.5)	0.51
Existence of medical professional among family or friends, n (%)	116 (41.9)	96 (33.3)	0.04
Oral health-related behaviors, n (%)			
Fluoride toothpaste	216 (78.0)	210 (72.9)	0.16
Mouthrinse	80 (28.9)	68 (23.6)	0.15
Dental floss or interdental brush	179 (64.6)	105 (36.5)	<0.01
Electric toothbrush	28 (10.1)	32 (11.1)	0.70
Tooth brushing frequency, n (%)			
Twice daily or more	255 (92.1)	263 (91.3)	0.75

HL, health literacy; IQR, interquartile ranges; SD, standard deviation

Multivariate logistic regression analysis of factors associated with preventive dental visits, HL, and oral-health behaviors is shown in Table 3. Students who visited a dentist for preventive care were more likely to exhibit higher total HL (adjusted odds ratios [aOR], 1.03; 95% confidence interval [CI], 0.99–1.07), have medical professionals among family or friends (aOR, 1.35; 95% CI, 0.94–1.93), and use fluoride toothpaste (aOR, 1.38; 95% CI, 0.92–2.08). However, no significant differences were observed between participants with and without preventive dental visits. On the other hand, students who visited a dentist for preventive care exhibited significantly higher rates of dental floss and interdental brush use compared to those who did not (aOR, 3.12; 95% CI, 2.19–4.45).

**Table 3:** Multivariate logistic regression analysis for association between preventive dental visit and oral-health behaviors (n=565)

	aOR	95% CI	p-value
Health literacy※	1.03	0.99–1.07	0.05
Sex			
Female	0.99	0.66–1.47	0.94
Existence of medical professional among family or friends	1.35	0.94–1.93	0.11
Oral health-related behaviors			
Fluoride toothpaste	1.38	0.92–2.08	0.12
Mouthrinse	1.08	0.72–1.62	0.70
Dental floss or interdental brush	3.12	2.19–4.45	<0.01
Electric toothbrush	0.85	0.48–1.50	0.57
Tooth brushing frequency			
Twice daily or more	0.94	0.50–1.78	0.85

※Health literacy changes by 1 unit; CI, confidence interval; aOR, adjusted odds ratio

## Discussion

This study revealed the following three findings: (1) An assessment of pharmacy students’ knowledge of OC prior to clinical training suggests that their knowledge regarding home healthcare and dysphagia/rehabilitation may be lacking. (2) Pharmacy students prior to clinical training score low on practical HL items relevant to clinical practice. (3) We suggest that preventive dental visit among Japanese pharmacy students is associated with improved rates of dental floss use. These findings may provide insights for addressing challenges in the teaching methods for OC and HL among pharmacy students in the future.

### Participant characteristics

This study focused on pharmacy students. The majority of participants were female, which is consistent with the high enrollment rate of females in Japanese pharmacy programs. In addition, the Japanese government has implemented the 8020 campaign, which aims to retain at least 20 natural teeth by the age of 80. however, only 58.3% of pharmacy students correctly understood the 8020 campaign, similar to general university students (59.2%) [23]. The awareness of oral hygiene among pharmacy students before clinical training may be similar that of general university students.

### Knowledge of OC and oral-health behaviors

This study suggests that Japanese pharmacy students acquire knowledge regarding dry mouth, stomatitis, periodontal disease, oral function, OC, and preventive dentistry within the existing curriculum. Furthermore, the correct answer rates tended to increase with grade level for items for which clinical information was crucial. However, the understanding was insufficient in areas related to home healthcare, such as the oral structure, dysphagia and rehabilitation, and OC assistance/nursing. In recent years, home healthcare and oral health support has been viewed as a domain in which pharmacists can leverage their professional competencies [24, 25]. Therefore, pharmacy students need education that enables them to practice OC that considers the patient’s condition, as described in “Practice of Drug Therapy” [6] in the “Model Core Curriculum for Pharmacy Education,” and in particular, OC education related to home healthcare may be necessary.

The “Model Core Curriculum for Pharmacy Education” also includes OC as an example of participation in activities that contribute to maintaining and improving the health of individual residents in the community, under the heading of “Contribution to Community Healthcare and Public Health.” [6]. Japanese drugstores carry various over-the-counter oral-hygiene products, such as rounded soft-bristle toothbrushes and fluoride toothpaste. Therefore, pharmacists have many opportunities for oral disease prevention and frontline assessment, including promoting the use of toothpaste, toothbrushes, and recommendations for dental visits [26]. However, to actively engage with users regarding oral hygiene, pharmacists need knowledge about oral health products such as dental floss, as well as skills to support users in improving their HL. A nationwide survey on oral assessment and healthcare in nursing schools reported that lectures on the relationship between periodontal disease and diabetes/cardiovascular disease, as well as fluoride-based caries prevention, were given to < 50% of the students, and dental floss usage was taught to only approximately 30% [27]. Many pharmacy students practiced brushing their teeth and using fluoride toothpaste in this study. However, whether these students, who practice these oral hygiene behaviors, can properly explain them to users remains unclear.

### HL

Pharmacy students prior to clinical training scored low on practical HL items relevant to clinical practice in this study. Among HC items, high scores were observed for “follow instructions on medication,” whereas low scores were observed for “understand what to do in a medical emergency” and “judge the advantages and disadvantages of different treatment options.” Although this reflects confidence in the knowledge acquired as pharmacy students, it also indicates limited clinical experience in handling sudden illnesses or selecting from multiple treatment options.

### Relationship between preventive dental visit and oral-health behaviors

The study revealed that pharmacy students who visited a dentist for preventive care were associated with a higher rate of dental floss use compared to those who did not. A survey conducted in Japan regarding interdental cleaning reported that the main factor encouraging respondents to commence interdental self-care was a clinical recommendation from dentists and dental hygienists [28]. Therefore, preventive dental visits may provide opportunities to receive regular oral health instruction from a dental hygienist and, pharmacy students may better understand the importance of using dental floss and interdental brushes and may be practicing these habits. Conversely, college students with favorable oral health behaviors may also be likely to visit dentists regularly [23]. Therefore, students with already established self-care habits may have been more likely to seek preventive dental care. In addition, students in the preventive dental visits group more frequently had medical professionals among family members or friends than those in the non-visit group. However, finally preventive dental visits were not associated with the presence of medical professionals among family or friends. Recently, information on health has been widely disseminated on television and the internet, facilitating convenient access [29]. Therefore, pharmacy students may now perceive preventive dental visits as more personally relevant.

Although students with preventive dental visits had higher total HL and HP HL scores than those without preventive dental visits, total HL was not independently associated with preventive dental visits after adjustment. Metanmo et al. reported a positive correlation between communicative HL and age [30]. Thus, a large percentage of university students responded that HL was difficult due to their limited experience with medical care and daily life, which may have influenced the results. On the other hand, HP-HL, which evaluates the ability to create or maintain a healthier living environment, may be less influenced by experience with medical care and daily life. In addition, pharmacy students’ self-efficacy improves through clinical training, which influences retention and reconstruction of their specialized pharmaceutical knowledge [31]. In this study, preventive dental visits did not differ significantly according to HC-HL and DP-HL among pre-clinical students. Therefore, if this study had included students after the completion of their clinical rotations, the results may have differed according to the students’ confidence and behavioral changes after their rotations.

### Limitations

This study had several limitations. First, knowledge of OC was evaluated using only 20 questions selected from 13 domains by faculty members in the School of Pharmacy. Therefore, item-selection bias and limited domain representativeness are possible. Since the measurement of OC knowledge was not based on a scale that has undergone internal validity testing, the evaluation method may not be optimal. Furthermore, it is not possible to verify the appropriateness of the selected areas or content. Therefore, an inherent limitation of this study is the absence of an established standardized method for measuring knowledge of OC. Thus, future research may need to develop educational scales for measuring pharmacy students’ knowledge of OC. Second, the participants were pharmacy students in their first to fourth years; thus, students with practical training were not included. Therefore, a discussion about a 6-year systematic education in OC for pharmacy students is challenging. This study discusses pharmacy students’ understanding of OC and their HL before clinical practice. Third, this study targeted pharmacy students from a single university, potentially introducing selection bias. Therefore, when interpreting the findings, limitations regarding generalizability must be considered, and future studies involving pharmacy students from other institutions are necessary. Fourth, cross-sectional studies cannot determine causality, and future longitudinal studies are required for the same. Finally, although students were informed that their academic performance and credit acquisition would not be affected, psychological pressure to respond may have influenced the relatively high response rate.

## Conclusions

This study suggests that pharmacy students before clinical practice may lack knowledge of home healthcare and dysphagia/rehabilitation of OC, and that their practical HL skills in clinical settings are low. These findings may provide insights for addressing challenges in the teaching methods for OC and HL among pharmacy students in the future. Additionally, it may be necessary to develop a scale to evaluate OC education among pharmacy students in Japan.

## Supplementary Information

Below is the link to the electronic supplementary material.

Supplementary Material 1

Supplementary Material 2

## Data Availability

The datasets used during this study are available from the corresponding author upon reasonable request.

## Abbreviations

CI: Confidence Interval
DP: Disease Prevention
HC: Healthcare
HL: Health Literacy
HLS-EU-Q47: The European Health Literacy Survey Questionnaire
HP: Health Promotion
OC: Oral Care
OR: Odds Ratios
